# SARS-CoV-2 Infection in San Patrignano, the Largest European Drug Rehabilitation Community

**DOI:** 10.3390/ijerph20032136

**Published:** 2023-01-24

**Authors:** Isabella Sala, Carlotta Micaela Jarach, Vincenzo Bagnardi, Maria Sofia Cattaruzza, Michela Morri, Paolo Ottogalli, Vincenzo Zagà, Silvano Gallus, Antonio Boschini

**Affiliations:** 1 Department of Statistics and Quantitative Methods, University of Milan-Bicocca, 20126 Milan, Italy; 2 Department of Medicine and Surgery, University of Milan-Bicocca, 20126 Milan, Italy; 3 Department of Environmental Health Sciences, Istituto di Ricerche Farmacologiche Mario Negri IRCCS, 20156 Milan, Italy; 4 Department of Public Health and Infectious Diseases, Sapienza University of Rome, 00185 Rome, Italy; 5 Società Italiana di Tabaccologia (SITAB), 00136 Rome, Italy; 6 Hygiene and Public Health Unit, AUSL della Romagna, 47924 Rimini, Italy; 7 San Patrignano Medical Center, 47853 Coriano, Italy

**Keywords:** SARS-CoV-2, COVID-19, hospitalization, Italy, mortality

## Abstract

Background: Studies on SARS-CoV-2 conducted in confined settings for prolonged times allow researchers to assess how the coronavirus spreads. San Patrignano (SP), Italy, is the largest European drug rehabilitation facility. Methods: Between 15 October and 31 December 2020, all SP residents were tested for SARS-CoV-2. We analyzed the relationships between individual characteristics and being SARS-CoV-2-positive. Three selected predictive models were used to calculate the number of expected hospitalizations. For each model, we summed the estimated individual risks to obtain the expected number of hospitalizations in our sample, and we tested whether the observed and expected numbers differed. Results: Of 807 residents, 529 (65.6%) were SARS-CoV-2-positive. Of these 323 (61.1%) were symptomatic. A strong relationship was found between being positive and living connections (*p*-value < 0.001). No statistically significant relationship was found with age, sex, smoking history, or comorbidities. Although 9 to 17 hospitalizations were expected, no hospitalizations were observed (*p*-value < 0.001). No one died of COVID-19. Conclusions: The peculiar characteristics of SP residents or the SP environment might at least partially explain the null hospitalization rates. Despite the extreme uniqueness of our population and despite the protected environment and all precautions that were taken, the fact that the virus was able to circulate and infect a large portion of the population highlights the fundamental role of social interactions in the spread of the disease.

## 1. Introduction

Controlling SARS-CoV-2 outbreaks in confined settings is challenging due to the difficulty in enforcing social distancing measures and quarantine. However, enclosed environments are ideal settings to study the spread of an infectious disease. One of the most striking case studies in this field was that of the Diamond Princess Cruise [1].

The studies that have focused on the expansion of SARS-CoV-2 in closed environments for prolonged periods were mainly conducted in nursing homes [2], where, however, the population is peculiar, as it is old and has particular comorbidities. There are also some, albeit few, studies that were conducted in prisons, which are likely populated by fragile people [3]. In fact, prisoners are at an extreme risk of contagion, given the overcrowding in prisons and the limited movement [4]. A 2020 study of prisons in Massachusetts showed that the incidence of COVID-19 among inmates was three times higher than in the general population living in the same state, findings that were similar to those in other European studies [5]. Specifically, within the Italian context [6], according to a study conducted on 7599 prisoners, more than 20% of the inmates became positive for SARS-CoV-2 between March 2020 and February 2021, the majority in the second wave (autumn and winter 2020). With reference to the second wave, the Italian article also reported the hospitalizations and deaths in three specific prisons (all around Milan). Out of 552 total SARS-CoV-2-positive inmates, 24 were hospitalized (4.3%) and 2 died (8.3% of those hospitalized) [6].

To our knowledge, no study has been conducted specifically in rehabilitation centers for former drug addicts. In terms of populations, these places could be comparable in some ways with prisons, but they retain certain peculiarities (i.e., they are not forcibly confined in cells) that make it necessary to carry out an accurate and precise analysis to confirm or deny what has been found for inmates.

Between October and December 2020, an outbreak of SARS-CoV-2 occurred in the largest European residential drug rehabilitation community. Our investigation specifically looked at a particular population (no one could smoke tobacco or drink alcohol). The objective of this study was to describe the spread of SARS-CoV-2 among the residents of this closed setting following any possible progression of COVID-19 during the early stages of the outbreak.

## 2. Materials and Methods

The target population was the residents of the community of “San Patrignano” (SP), a private residential center for the rehabilitation of drug users in Coriano, Italy, the largest community of its kind in Europe. According to SP regulations, substance use, including alcohol and tobacco, is not allowed. All drug users who enter the community receive drug-free therapy, which lasts for a mean of 30 months. Opioid agonist treatment can be used only in the early detoxification phase. The physical property covers 1000 acres and consists of several residential buildings as well as extensive pastoral and agricultural lands. SP residents live and work together, sharing dormitories, workplaces, and recreational and leisure facilities. The community is economically self-sufficient, with each resident contributing to the institutional activities based on his or her skills and physical capacity. A medical center, which includes outpatient and inpatient facilities, a laboratory for routine analyses, and a radiology service, cares for and treats all residents of the community (www.sanpatrignano.org/la-comunita/, accessed on 12th January 2023).

Since the first case of SARS-CoV-2 infection was reported in Italy on 21 February 2020, all residents (i.e., recovering addicts, resident staff, founders, educators, and respective families) were confined, whereas all external employees stayed home on paid leave. Infection prevention strategies, such as the use of personal protective equipment (PPE), were implemented by essential personnel (e.g., educators, doctors, and nurses). Despite the precautionary measures that were adopted, the first case of SARS-CoV-2 infection was detected on 18 October 2020, and the virus began spreading throughout SP. In response to this first case of SARS-CoV-2 infection, the following health interventions were implemented to prevent further viral spread: mass screening of all residents; isolation of positive cases until obtaining negative test results; quarantining close contacts of SARS-CoV-2 cases for 10 days (with a control swab on the 10th day); molecular testing of all individuals with respiratory, gastrointestinal, or general symptoms; separation of the different work areas of the community (i.e., avoid contact among residents who work in different areas); and closure of common areas, such as dining rooms, theatres, and gyms. In the post-epidemic phase, serological tests were conducted on individuals who had no symptoms or who had tested negative when using molecular swabs in the mass screenings.

Moreover, a large area of the SP community that included dormitories and recreational settings was designated for infected individuals only. In this fashion, positive cases were isolated from non-infected residents, but they were at lower risk of experiencing severe psychological stress due to isolation.

This retrospective observational study included all 807 former drug users living in SP during the outbreak period from 15 October to 31 December 2020. We excluded from the analyses the AIDS patients hospitalized in the SP medical center who were isolated from the other residents. All included subjects were tested for SARS-CoV-2, and the dates of the first positive tests of confirmed cases were collected. A confirmed case of SARS-CoV-2 infection was defined as a resident of the community of SP with a positive serological or molecular test. At the beginning of the outbreak, symptomatic cases were tested via a molecular swab, as were their close contacts (those who slept in the same environment). Since the beginning, a screening was carried out on all guests and repeated once a month (three times in total). Once the outbreak was over, a serological screening was performed but only on those who had never tested positive.

Patients entering San Patrignano signed an informed consent for the management of their routine health care data. Given the observational nature of the present study, no ethical approval was requested. For these reasons, the present study was performed in accordance with the relevant guidelines and Italian regulations and legislation.

The daily numbers of SARS-CoV-2 cases were plotted by the date of the first positive test. The epidemic curve was fitted using a 7-day moving average.

Differences in the distributions of demographic and clinical characteristics between subjects with and without confirmed infections were evaluated using Student’s t-test for continuous variables and the Pearson chi-square test for categorical variables [7]. To calculate the expected number of hospitalizations among people who tested positive for SARS-CoV-2, the risk of hospitalization (p_i_) was estimated for each infected subject i as follows. Individual risk estimates were calculated using three different published predictive models: (i) The Cleveland Clinic (Cleveland Clinic, OH, USA) model [8], which includes both sociodemographic and clinical features as covariates, such as age, ethnicity, sex, BMI, smoking status, comorbidities, and symptoms. The model was built and validated using a total of 4536 patients who tested positive at the Cleveland Clinic’s hospitals and outpatient locations in Ohio and Florida. We derived the model coefficients to estimate the individual hospitalization risks from the online version of the predictive model, which is available at https://riskcalc.org/COVID19Hospitalization/ (accessed on 1st April 2022). (ii) The model by Karaismailoglu et al. [9] was based on data from 979,430 confirmed cases of SARS-CoV-2 that were made publicly available by the Mexican Ministry of Health. The model includes sociodemographic (i.e., age, sex, and smoking) and clinical (i.e., pneumonia, chronic kidney failure, chronic obstructive pulmonary disease, diabetes, hypertension, cardiovascular disease, obesity, immunosuppression, and asthma) covariates. Because an online calculator was not available, we used a validated web-based tool (WebPlotDigitizer, https://automeris.io/WebPlotDigitizer, accessed on 16 January 2023) to extract the probability of being hospitalized from the published graphical representation of the model. (iii) Nyberg et al. [10] computed age-specific absolute risks using data from 246,869 patients with confirmed SARS-CoV-2 infections tested at three laboratories in England that receive specimens from nationwide testing.

For each model, we summed the estimated individual risks (p_i_) to obtain the expected number of hospitalizations in our sample, and we tested whether the observed and expected numbers differed using an exact Poisson test [11].

Furthermore, we plotted a network graph to visualize the spread of SARS-CoV-2 in the community of SP. In this network, nodes represent each subject, and links between nodes were created when subjects were connected by living and/or working areas (triggers). First, we constructed a matrix where diagonal elements were the number of triggers in which each subject participated (i.e., two triggers for each subject: living and working areas), and off-diagonal elements were the number of triggers that each pair of subjects shared. Cohesion metrics such as the average degree (i.e., the average number of links per node in the graph) and network density (i.e., the proportion of potential links in the network that are actual links) were calculated [12]. Then, we visualized the network using the R package igraph [13]. All statistical analyses were performed using SAS software version 9.4 and R software version 3.6.0.

## 3. Results

**Figure 1:**
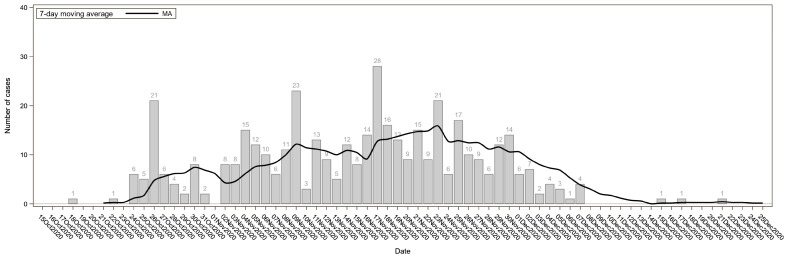
Epidemic curve showing daily SARS-CoV-2 cases and 7-day moving average (black line) in the Community of “San Patrignano”, a drug rehabilitation community in Italy, from October to December 2020.

Before entering SP, the majority of the residents were using multiple drugs simultaneously (polysubstance dependence), although they were not necessarily dependent on all the drugs used. In the population that was considered (N = 807), the main dependencies were cocaine/crack (65%, although the use involved more than 95% of the sample), followed by heroin (59.7%), alcohol (31.9%), and finally cannabis (used by almost everyone but causing dependence in 8% of the sample). It is important to note how many were using drugs through injection (PWID: people who inject drugs and NIDU: non-injecting drug users). Between 15 October and 31 December 2020, out of 807 recovering addicts living in SP, 529 (65.6%) tested positive for the SARS-CoV-2 infection: 428 (80.9%) had a positive molecular test, and 101 (19.1%) had a positive serological test. The distribution of SARS-CoV-2 cases over time is shown in Figure 1. There were no statistically significant differences between infected and non-infected subjects with respect to demographics, smoking habits, and clinical characteristics. A statistically significant relationship with SARS-CoV-2 infection was found for living areas (*p*-value < 0.001) (Table 1). Among the 529 infected subjects, 323 (61.1%) were symptomatic cases. Table 2 shows the frequencies of different symptoms among the symptomatic cases. The most common symptoms were low-grade fever (44.6%), fever (24.8%), and anosmia (21.7%).

None of the subjects who tested positive were hospitalized. According to the three different models considered for the estimate of hospitalization risk, we would have expected 9.3 (1.8%) hospitalized subjects, according to the Cleveland Clinic model [8]; 12.2 (2.3%), according to the age-specific absolute risks reported by Nyberg et al. [10]; and 16.8 (3.2%), according to the model by Karaismailoglu et al. [9]. All differences between the observed and expected values were statistically significant (*p*-value < 0.001).

**Figure 2:**
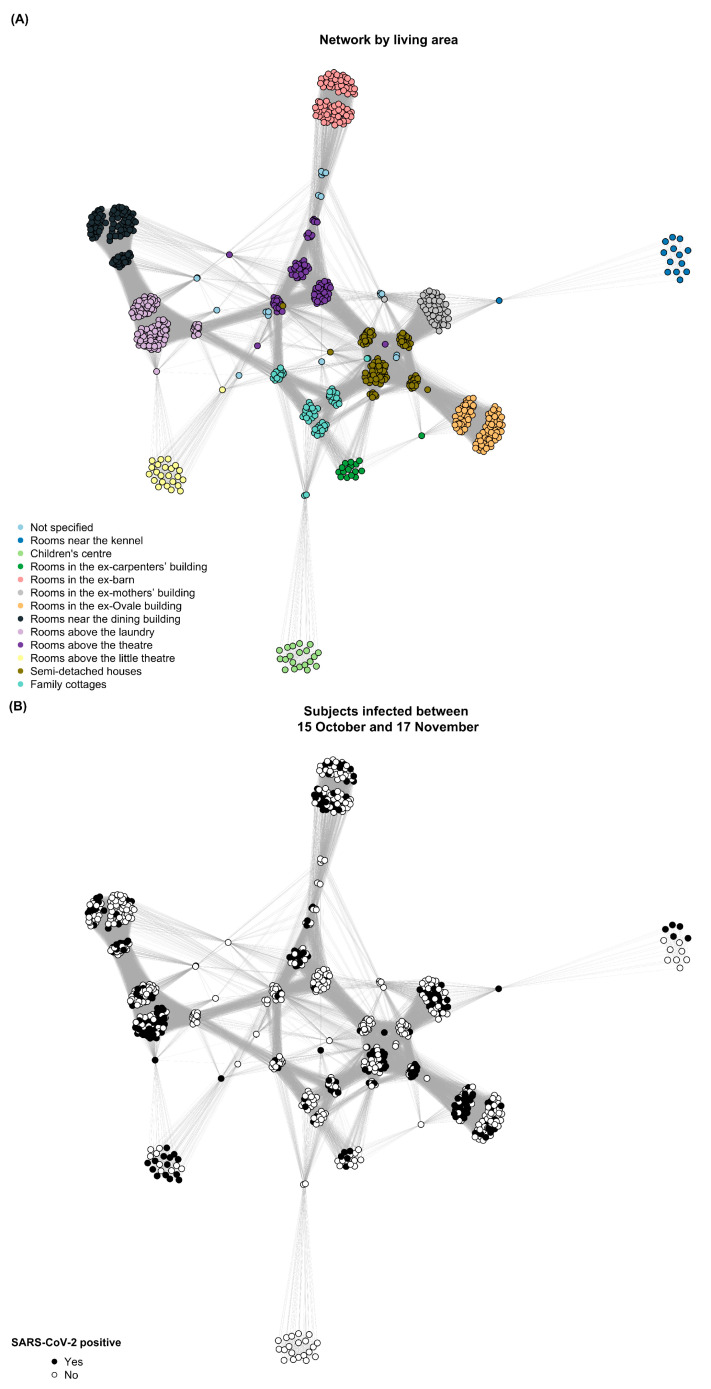

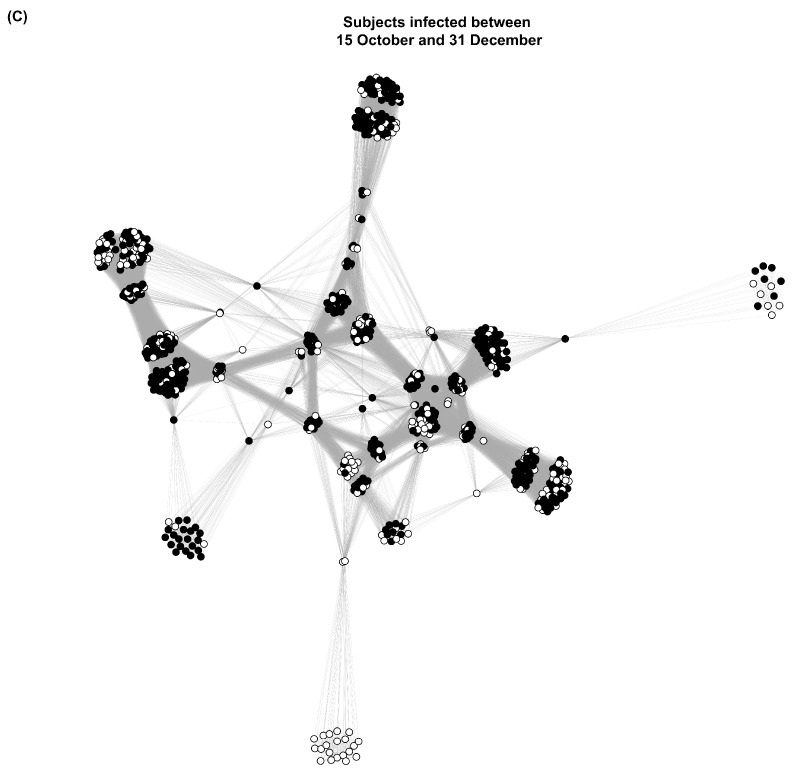
Network analysis graphs of SARS-CoV-2 transmission in the Community of “San Patrignano”. Links between subjects (nodes) are given by living and working areas. Panel (**A**) shows the network by living areas. In panel (**B**), the individuals who became infected between the outbreak onset (15 October 2020) and the observed peak (17 November 2020) are shown in black. In panel (**C**)**,** all individuals who became infected by 31 December 2020 are shown in black.

Figure 2 depicts the progression of virus transmission within the community. The network is represented in three phases of the outbreak: before the first case was detected, the day when the highest number of daily infections was reached, and after the last case was observed. The virus spread widely among strongly linked subjects (e.g., those with rooms above the laundry and rooms in the ex-Ovale building), whereas it was contained or did not circulate in more isolated clusters (e.g., the children’s center and family cottages). The network graph contained 42,556 weighted edges, leading to an average degree of 52.73 and a network density of 0.13.

## 4. Discussion

To our knowledge, this is the first investigation of SARS-CoV-2 spread and hospitalization rates among former drug addicts in a closed rehabilitation community. This Italian study was conducted during the second wave of the COVID-19 pandemic (autumn 2020) and found that although two out of three SP residents tested positive for SARS-CoV-2 in less than 3 months, none of them were hospitalized—contrary to expectations—and most importantly no one died. No relationships were found between SARS-CoV-2 infection and demographics, smoking history, or clinical characteristics. On the contrary, great importance was placed on the prolonged social interactions among individuals. In fact, the network analysis metrics suggest that SP residents were strongly linked with each other, making it harder to control the spread of the virus. We observed that in some living areas the virus did not spread (i.e., the children’s center) or disseminated among a subgroup before spreading to the other people sharing the living area (e.g., the rooms above the laundry and the semi-detached houses). Contacts made at work were likely to play an important role in the virus’s pathway. As a result, people who lived and worked in the same area were more likely to become infected at the same time and then spread the virus to their roommates.

The emphasis on close contacts as a means of rapid virus expansion is also a characteristic of the evidence on outbreaks within a prison context, which remains dissimilar to SP’s reality but homologous and comparable for some characteristics [14,15,16].

Our study makes an important contribution to the discussion about COVID-19 and confined settings, not only because we were able to test the entire population of interest but particularly because the SP population is extremely unique. SP residents are relatively young, and despite the large majority being former tobacco smokers or former alcohol drinkers, they follow the strict SP rules. They cannot smoke tobacco or drink alcohol anymore when entering the community. They cannot be assimilated into the general population; therefore, it was understandable to expect different rates of hospitalization and death compared to those living in the same area outside the community. However, it would have been plausible to find data similar to what has been published in the literature on inmates. In future research, it may be necessary to compare SP residents with even more similar populations abroad.

Despite the scarcity of research to compare with SP residents, our findings highlight the relevance of proximity as a factor in epidemic prevention, particularly in closed settings such as recovery communities.

The reasons why there were far fewer hospitalizations than expected were not completely clarified. One possibility could be the unique characteristics of SP residents, namely the fact that there are no current smokers or current alcohol drinkers. Additionally, SP, as a physical place, might have played a protective role. The facility was a particularly healthy environment (e.g., it is located in a rural setting exposed to a low level of air pollution), as residents spent a lot of time outdoors taking part in manual activities. Finally, the isolated context might have led to a lower exposure to other pathogens that could negatively influence the progression of COVID-19.

## 5. Conclusions

In conclusion, in this population, although isolated, the virus was nonetheless able to circulate, and in fact two thirds of the SP residents were infected but without an unfavorable burden or mortality. As found elsewhere, physical interactions played an important role in SP in terms of the spread of the epidemic. In other closed environments such as prisons, high transmissibility was found, but no excess risk of hospitalization, severity, or mortality compared to the general population was observed. Our data do not allow us to draw any conclusions about the cause of our population’s lower burden of disease, leading us to speculate that there may be some other unobserved characteristics that distinguish this population. However, despite the extreme uniqueness of our population and despite the protected environment and all precautions that were taken, the fact that the virus was able to circulate and infect a large portion of the population highlights the fundamental role of social interactions in the spread of the disease.

## Figures and Tables

**Table 1:** Demographic and clinical characteristics of former drug users living in the Community of “San Patrignano” according to SARS-CoV-2 infection status.

Characteristics	Total N	SARS-CoV-2-Positive Subjects N (%)	*p*-Value (SARS-CoV-2-Positive vs. -Negative Subjects)
Total	807	529 (65.6)	
Sex			
Women	158	94 (59.5)	0.074
Men	649	435 (67.0)	
Age (in years)			
<30	341	218 (63.9)	0.189
30–39	318	220 (69.2)	
≥40	148	91 (61.5)	
Living area ^a^			
Living area 1	195	110 (56.4)	0.001
Living area 2	293	198 (67.6)	
Living area 3	300	215 (71.7)	
Not specified	19	6	
Working area ^b^			
Working area 1	22	0 (0.0)	0.559
Working area 2	22	4 (18.2)	
Working area 3	29	14 (48.3)	
Working area 4	13	7 (53.8)	
Smoking history			
Yes	786	515 (65.5)	0.913
No	21	14 (66.7)	
Pack Years, mean (SD)	14.8 (10.8)	14.5 (10.0)	0.369
Comorbidities ^c^			
Yes	111	78 (70.3)	0.260
No	696	451 (64.8)	

^a^ Living areas were categorized as follows:- living area 1: children’s center, family cottages, and semi-detached houses; living area 2: rooms in the ex-carpenters’ building, rooms near the kennel, rooms near the dining building, rooms in the ex-mothers’ building, rooms above the little theatre, and rooms above the theatre; living area 3: rooms in the ex-Ovale building, rooms above the laundry, and rooms in the ex-barn. ^b^ Working areas were categorized as follows: working area 1: children’s center, direction, reception, graphic design, and medical center; working area 2: weaving, building, decoration, laundry, electricians, and plumbers; working area 3: kennel, animal farming, garden, farm, and warehouse; working area 4: cheese factory, kitchen, bakery, and butcher. ^c^ The following comorbidities were taken into consideration: obesity (*n* = 87), hypertension (*n* = 8), neoplasia (*n* = 13), diabetes (*n* = 6), and liver cirrhosis (*n* = 4).

**Table 2:** Symptoms among 323 subjects with symptomatic SARS-CoV-2 infection in the Community of “San Patrignano”.

Symptoms	SARS-CoV-2-Positive Subjects with Symptoms	
	N	%
Anosmia	70	21.7
Respiratory symptoms ^a^	11	3.4
Nausea	5	1.5
Diarrhea	4	1.2
Headache	16	5.0
Low-grade fever	144	44.6
Fever	80	24.8
Hyperpyrexia	24	7.4
Pain	25	7.7
Others	12	3.7

^a^ Respiratory symptoms included rhinitis, cough, cold, sore throat, and pharyngodynia.

## Data Availability

The data presented in this study are available on request from the corresponding author. The data are not publicly available, as to the study is still ongoing.
